# Modulation of Pro-Oxidant and Pro-Inflammatory Activities of M1 Macrophages by the Natural Dipeptide Carnosine

**DOI:** 10.3390/ijms21030776

**Published:** 2020-01-25

**Authors:** Claudia G. Fresta, Annamaria Fidilio, Giacomo Lazzarino, Nicolò Musso, Margherita Grasso, Sara Merlo, Angela M. Amorini, Claudio Bucolo, Barbara Tavazzi, Giuseppe Lazzarino, Susan M. Lunte, Filippo Caraci, Giuseppe Caruso

**Affiliations:** 1 Ralph N. Adams Institute for Bioanalytical Chemistry, University of Kansas, Lawrence, KS 66047-1620, USA; forclaudiafresta@gmail.com (C.G.F.); slunte@ku.edu (S.M.L.); 2 Department of Pharmaceutical Chemistry, University of Kansas, Lawrence, KS 66047-1620, USA; 3 Department of Drug Sciences, University of Catania, 95125 Catania, Italy; afunict@gmail.com (A.F.); grassomargherita940@gmail.com (M.G.); carafil@hotmail.com (F.C.); 4 UniCamillus—Saint Camillus International University of Health Sciences, 00131 Rome, Italy; giacomo.lazzarino@unicamillus.org; 5 Bio-nanotech Research and Innovation Tower (BRIT), University of Catania, 95125 Catania, Italy; nmusso@unict.it; 6 Oasi Research Institute—IRCCS, 94018 Troina (EN), Italy; 7 Department of Biomedical and Biotechnological Sciences, University of Catania, 95125 Catania, Italy; sara_merlo@hotmail.com (S.M.); amorini@unict.it (A.M.A.); claudio.bucolo@unict.it (C.B.); 8 Institute of Biochemistry and Clinical Biochemistry, Catholic University of Rome, 00168 Rome, Italy; barbara.tavazzi@unicatt.it; 9 Fondazione Policlinico Universitario A. Gemelli IRCCS, 00168 Rome, Italy; 10 Department of Chemistry, University of Kansas, Lawrence, KS 66047-1620, USA

**Keywords:** carnosine, M1 macrophages, nitric oxide, oxidative stress, inflammation, antioxidants, energy metabolism

## Abstract

Carnosine is a natural endogenous dipeptide widely distributed in mammalian tissues, existing at particularly high concentrations in the muscles and brain and possesses well-characterized antioxidant and anti-inflammatory activities. In an in vitro model of macrophage activation, induced by lipopolysaccharide + interferon-gamma (LPS + IFN-γ), we here report the ability of carnosine to modulate pro-oxidant and pro-inflammatory activities of macrophages, representing the primary cell type that is activated as a part of the immune response. An ample set of parameters aimed to evaluate cytotoxicity (MTT assay), energy metabolism (HPLC), gene expressions (high-throughput real-time PCR (qRT-PCR)), protein expressions (western blot) and nitric oxide production (qRT-PCR and HPLC), was used to assess the effects of carnosine on activated macrophages challenged with a non cytotoxic LPS (100 ng/mL) + IFN-γ (600 U/mL) concentration. In our experimental model, main carnosine beneficial effects were: (1) the modulation of nitric oxide production and metabolism; (2) the amelioration of the macrophage energy state; (3) the decrease of the expressions of pro-oxidant enzymes (Nox-2, Cox-2) and of the lipid peroxidation product malondialdehyde; (4) the restoration and/or increase of the expressions of antioxidant enzymes (Gpx1, SOD-2 and Cat); (5) the increase of the transforming growth factor-β1 (TGF-β1) and the down-regulation of the expressions of interleukins 1β and 6 (IL-1β and IL-6) and 6) the increase of the expressions of Nuclear factor (erythroid-derived 2)-like 2 (Nrf2) and heme oxygenase-1 (HO-1). According to these results carnosine is worth being tested in the treatment of diseases characterized by elevated levels of oxidative stress and inflammation (atherosclerosis, cancer, depression, metabolic syndrome, and neurodegenerative diseases).

## 1. Introduction

Different types of cells are involved in the innate immune response, with macrophage cells representing those primarily activated [1,2], especially under several conditions characterized by oxidative stress and inflammation [3,4]. Experimentally, both oxidative stress, obtained by stimulation with phorbol 12-myristate 13-acetate (PMA) [5,6], and inflammation, triggered by using a combination of lipopolysaccharides (LPS) and interferon-gamma (IFN-γ) [7], can successfully be achieved in macrophages. Depending on the cellular environment and the stimulation signals, activated macrophages give rise to a plethora of phenotypes, described by M1 (classically activated macrophages), M2 (alternatively activated macrophages), and their respective sub-groups [8]. The M1 phenotype is mainly involved in the production of pro-inflammatory cytokines as well as reactive oxygen (ROS) and nitrogen (RNS) species, while the M2 phenotype is primarily associated with the production of anti-inflammatory cytokines and proliferative agents, finalized to wound healing and tissue repair [9]. Nuclear factor (erythroid-derived 2)-like 2 (Nrf2) as well as its downstream gene heme oxygenase 1 (HO-1) are directly involved in the detoxification from ROS and RNS and in the anti-inflammatory response [10]. With regard to macrophage behavior, the induction of HO-1 has been shown to determine the switch from M1 to M2 phenotype [11], also regulating the cytoprotective activity of nitric oxide (NO) [12].

Stimulated macrophages are particularly active in generating NO. Physiologically, NO is a fundamental signaling gaseous molecule in the nervous, immune, and cardiovascular systems [13], transmitting both intracellular and intercellular signals crucial for cell and organ survival [13,14]. Among its numerous functions, NO participates to the regulation of brain glutamate metabolism [15], it is implicated in the modulation of a vascular tone [13] and it plays an important role in the integration of host defense, and regulation of inflammation in macrophages, being able to switch the macrophage phenotype from an activated cell to a cell susceptible to programmed cell death [14]. On the another hand, NO is recognized as one of the main signaling molecules involved in the inflammatory processes [15]; in fact, the inducible isoform of nitric oxide synthase (iNOS), overexpressed in macrophages and some other cell types under a variety of acute and chronic inflammatory conditions [16], is responsible of an intense production of NO, generally accompanied by increased ROS formation. The intrinsic risk in the temporal coincidence of the two phenomena is that superoxide anions easily react with NO producing peroxynitrite [17,18], an extremely reactive and toxic molecule able to damage the four major classes of biological macromolecules (DNA, carbohydrates, fatty acids and proteins,) and mitochondria [19]. The concomitant increased generations of RNS and ROS triggers a phenomenon commonly termed as oxidative/nitrosative stress, which is often encountered in various pathological conditions (acute and chronic neurodegenerative disorders, cancer and cardiovascular diseases).

Recently, it has been demonstrated that the uptake of carnosine (β-alanyl-L-histidine), a natural dipeptide widely distributed in mammalian tissues [20], is highly increased in macrophages under pro-inflammatory conditions [21]. In vivo experiments showed that carnosine is involved in the regulation of macrophage function, increasing their phagocytic activity and ROS production [22]. Additionally, carnosine has been shown to decrease both oxidative stress and inflammation in an in vitro model of amyloid-induced inflammation [23]. Physiologically, carnosine is synthesized from the amino acids β-alanine and L-histidine in an ATP-dependent reaction catalyzed by the enzyme carnosine synthetase 1 [24,25]. This dipeptide is present at high concentrations in the mammalian brain (micromolar to millimolar range) [26,27], but it reaches its maximal concentrations in cardiac and skeletal muscles (up to 20 mM) [28]. Furthermore, strong evidence that carnosine protects against pathologies characterized by oxidative stress and/or inflammation such as diabetes [29], depression [30], cerebral ischemia [31] and Alzheimer’s disease (AD) [32] has been proved. In previous studies, we showed that 20 mM carnosine is highly effective to positively regulating macrophage and microglial functions [23,33]. In particular, we found that carnosine, in macrophages challenged with LPS + IFN-γ, increases the rate of NO transformation into its stable end-product nitrite, modulates the composition of macrophage sub-populations and decreases the release of pro-inflammatory cytokines into the cell medium.

In the present study, using pro-inflammatory conditions that did not cause an increase in cellular mortality, we evaluated the ability of carnosine to modulate NO metabolism as well as the variation of parameters representative of cellular energy metabolism, oxidative stress and inflammation in activated (M1) RAW 264.7 cells. Furthermore, in order to better understand the molecular mechanisms underlying carnosine effects, the protein expression levels of Nrf2 and HO-1, two key proteins regulating cellular responses to oxidative stress and inflammation, were examined.

## 2. Materials and Methods

### 2.1. Materials and Reagents

All chemicals were of analytical grade and purchased from Sigma (St. Louis, MO, USA) or Thermo Fisher Scientific Inc. (Pittsburgh, PA, USA) unless specified otherwise. RAW 264.7 cells (ATCC^®^ TIB-71™), Dulbecco’s modified Eagle medium (DMEM), fetal bovine serum (FBS) and penicillin/streptomycin antibiotic solution were purchased from American Type Culture Collection (ATCC, Manassas, VA, USA). Interferon-γ (IFN-γ), mouse recombinant, *Escherichia coli* was supplied by Calbiochem (Gibbstown, NJ, USA). Centrifuge tubes equipped with 3 kDa molecular weight cut-off filters, water, methanol, far-UV acetonitrile and chloroform (all HPLC-grade) were supplied by VWR International (West Chester, PA, USA). DAF-FM DA probe was purchased from Life Technologies (Carlsbad, CA, USA). C-Chip disposable hemocytometers were purchased from Bulldog Bio, Inc. (Portsmouth, NH, USA). A geNorm Housekeeping Gene Selection Kit was obtained by Primer Design Ltd. (Southampton, UK). QuantiTect SYBR Green PCR Kit, RNeasy Mini Kit, RNase-free DNase Set and QuantiTect Primer Assays were purchased from Qiagen (Hilden, Germany), while 18S rRNA primers (forward: 5ʹ-AGT CCC TGC CCT TTG TAC ACA-3ʹ; reverse: 5ʹ-GAT CCG AGG GCC TCA CTA AAC-3ʹ) were purchased by Eurofins MWG Synthesis GmbH (Ebersberg, Germany). Anti-GAPDH primary antibody was obtained from Millipore (Burlington, MA, USA); anti-Nrf2 primary antibody was obtained from Cell Signaling Technology Inc. (Danvers, MA, USA) and anti-HO-1 primary antibody was purchased from Abcam (Cambridge, UK). Secondary goat anti-rabbit labeled with IRDye 680 and goat anti-mouse labeled with IRDye 800 were purchased from Li-COR Biosciences (Lincoln, NE, USA). 384-well plates were obtained by Roche Molecular Systems Inc. (Pleasanton, CA, USA). Eppendorf LoBind 1.5 mL Microcentrifuge Tubes PCR Clean and PCR tubes were obtained from Eppendorf (Hamburg, Germany). A Sylgard 184 polydimethylsiloxane (PDMS) prepolymer and curing agent were obtained from Ellsworth Adhesives (Germantown, WI, USA).

### 2.2. Cell Culture and Treatment Protocol

The specific conditions employed to culture and maintain RAW 264.7 cells are the same previously described in details [33]. On the day of the experiment, cells were harvested, an aliquot of the cell suspension was used for cell counting (performed by using a C-Chip disposable hemocytometer and the trypan blue solution) and it was plated in polystyrene culture flasks or Petri dishes at the appropriate density. LPS (1 mg/mL) and IFN-γ (200,000 U/mL) stock solutions were prepared as previously described [34]. On the day of the experiment, once the cells adhered to the flask or Petri dish surface, cells were left untreated (resting control cells), treated with a combination of LPS + IFN-γ, or pre-treated (1 h) with carnosine and then subjected to pro-inflammatory stimulation [33]. Cells were then incubated for 6 or 24 h in a humidified environment at 37 °C and 5% CO_2_.

Supplementary Figure S1 depicts the experimental design employed to study carnosine effect on stimulated activated RAW 264.7 macrophages along with representative images showing the changes in cell morphology due to the M1-induced stimulation.

### 2.3. Cell Viability Measurement by MTT Assay

Cell viability of RAW 264.7 plated in 48-well plates (2.5 × 10^5^ cells/well) under our different experimental conditions was measured by the MTT assay as previously described [35,36]. Briefly, after the stimulation process, in the absence or in the presence of carnosine, MTT solution (1 mg/mL in DMEM medium) was added to each well followed by an incubation for 2 h at 37 °C. The formed crystals were melted with DMSO and used to read the absorbance at 569 nm using a microplate reader (Molecular Devices, Spectra Max M5, Sunnyvale, CA, USA). Cell viability data are expressed as the percent variation with respect to the absorbance at 569 nm recorded in untreated cells.

### 2.4. HPLC Analysis of Metabolites Representative of Cellular Energy Metabolism, Oxidative Stress, and Inflammation

RAW 264.7 macrophages were plated at the density of 4.5 × 10^6^ cells/Petri dish and stimulated as described in Section 2.2. Macrophages were deproteinized according to the organic solvent deproteinization, suitable to measure acid labile and easily oxidizable compounds [37]. The analytes of interest were separated and quantified by the ion-pairing HPLC method described in detail elsewhere [38]. The identification and quantification of the different compounds of interest in chromatographic runs of macrophage extracts were performed by comparing retention times, absorption spectra and the area of the peaks of chromatographic runs of mixtures containing known concentrations of ultrapure true standard mixtures.

### 2.5. Nitrite Determination Using the Griess Assay

The Griess assay was performed as previously described [33]. Briefly, RAW 264.7 macrophages were challenged for 24 h with LPS + IFN-γ (100 ng/mL + 600 U/mL, respectively), in the absence or in the presence of carnosine (20 mM). At the end of the stimulation protocol, 100 μL of supernatant were taken from each well and added to an equal volume of Griess reagent. After 15 min at room temperature in the dark, the absorbance was measured at 540 nm using a Varioskan^®^ Flash spectrophotometer (Thermo Fisher Scientific, Waltham, MA, USA). A nitrite standard calibration curve was prepared using nitrite standards (from 1 to 100 μM).

### 2.6. Western Blot Analysis

Western blot analysis were carried out as previously described [39] on RAW 264.7 macrophages harvested at 4 °C in RIPA buffer in the presence of a cocktail consisting of protease inhibitors, serine/threonine phosphatase inhibitors, and tyrosine protein phosphatase inhibitors. Each cell lysate was sonicated and then subjected to centrifugation followed by supernatant collection. The protein concentration in each sample was determined by Bradford’s reagent and by measuring the absorbance at 595 nm with a Varioskan^®^ Flash spectrophotometer (Thermo Fisher Scientific). Bovine serum albumin was used to build a standard curve. After a blocking step, membranes were incubated overnight at 4 °C with the selected primary antibodies (1:2000 for GAPDH; 1:1000 for all other primary antibodies). After three washing steps, membranes were incubated for 1 h at room temperature with secondary goat anti-rabbit labeled with IRDye 680 (1:15,000) and goat anti-mouse labeled with IRDye 800 (1:15,000). The Odyssey Infrared Imaging System (LI-COR Biosciences, Lincoln, NE, USA) was used for the detection of the hybridization signals. Western blot data were quantified by densitometric analysis (Image J software) in three to four different blots per experiment.

### 2.7. Gene Expression Analysis by Quantitative Real-Time PCR (qRT-PCR)

The concentration of total RNA, recovered from 4.5 × 10^5^ control cells or cells treated with LPS + IFN-γ, without or with 1 h pre-treatment with carnosine for 6 or 24 h, was determined by measuring the absorbance at 260 nm with a NanoDrop^®^ ND-1000 (Thermo Fisher Scientific, Waltham, MA, USA). The protocol employed for reverse transcription, sample amplification, fluorescence data collection, and sample quantification is the same previously described by us [23], with slight modifications. Briefly, the mRNA extracted (RNeasy Mini Kit) from each sample (1 µg) was retrotranscripted (SuperScript III First-Strand Synthesis SuperMix kit) and the resultant cDNAs, loaded on a 384-well plate, were amplified by using specific, genomewide, bioinformatically validated primer sets. Table 1 reports the official name, official symbol, alternative titles/symbols, detected transcript, amplicon length and primers catalogue number of all the QuantiTect Primer Assays (genomewide, bioinformatically validated primer sets) employed for the gene expression analysis.

Each sample amplification consisted of a total reaction volume of 10 μL (5 μL PCR Master Mix + 1 μL specific primers + 4 μL of cDNA (50 ng)). Amplification conditions and fluorescence data collection included: one cycle at 95 °C for 15 min, 50 cycles at 94 °C (15 s each), one annealing step at 56 °C for 30 s, and one cycle at 72 °C for 30 s. The negative control consisted of a reaction in absence of cDNA (5 μL PCR Master Mix + 1 μL specific primers + 4 μL of Tris-EDTA buffer) indicated as NTC (no template control). The relative RNA expression level for each sample was calculated using the 2^−ΔΔCT^ method (threshold cycle (CT) value of the gene of interest vs. CT value of the housekeeping gene) [40,41]. For accurate gene expression measurements with qRT-PCR, the results were normalized to the GAPDH housekeeping gene, selected using the geNorm Housekeeping Gene Selection Kit.

### 2.8. Statistical Analysis

Statistical analysis was performed using Graphpad Prism (Graphpad software, version 6, San Diego, CA, USA). The within group comparison was performed by the one-way analysis of variance (ANOVA). The post hoc Tukey test was used for multiple comparisons. Only two-tailed *p*-values less than 0.05 were considered statistically significant.

## 3. Results

### 3.1. LPS + IFN-γ Stimulation do not Change Cell Viability in RAW 264.7 Macrophages

**Figure 1:**
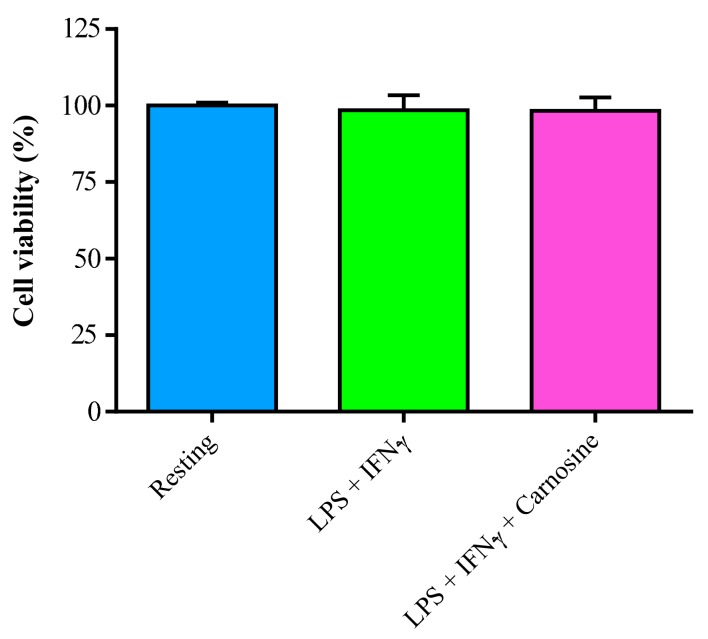
Cell viability in resting (control) RAW 264.7 macrophages and in RAW 264.7 macrophages stimulated with LPS (100 ng/mL) + IFN-γ (600 U/mL), in the absence or in the presence of carnosine (20 mM). Data are the mean of five independent experiments. Values were normalized with respect to control untreated macrophages (resting) and are expressed as the percent variation of cell viability.

In order to exclude any relevant toxic effect caused by the exposure to pro-inflammatory conditions, cell viability in RAW 264.7 macrophages, stimulated with a combination of LPS + IFN-γ, without and with 20 mM carnosine, was evaluated after 24 h incubation. As shown in Figure 1, cells receiving any of the two treatments had viability equal to that of resting (untreated) cells.

This indicates that any further metabolic, gene and protein expression changes are not linked to cytotoxic effects of LPS + IFN-γ, at least at the concentrations and time of incubation chosen in our experimental conditions.

Therefore the subsequent experiments were finalized to determine whether: (1) the absence of toxicity under M1 polarization was connected to any variation of different cellular biochemical functions related to oxidative/nitrosative stress, energy metabolism, and inflammation and (2) the natural dipeptide carnosine had significantly beneficial immuno-regulatory effects.

### 3.2. Carnosine Increases the Rate of Degradation of NO into NO_2_^−^ with no Inhibition of iNOS Activity

We have previously shown that carnosine, but not its constituting amino acids (β-alanine and L-histidine), is able to modulate NO in stimulated murine RAW 264.7 macrophages [33].

**Figure 2:**
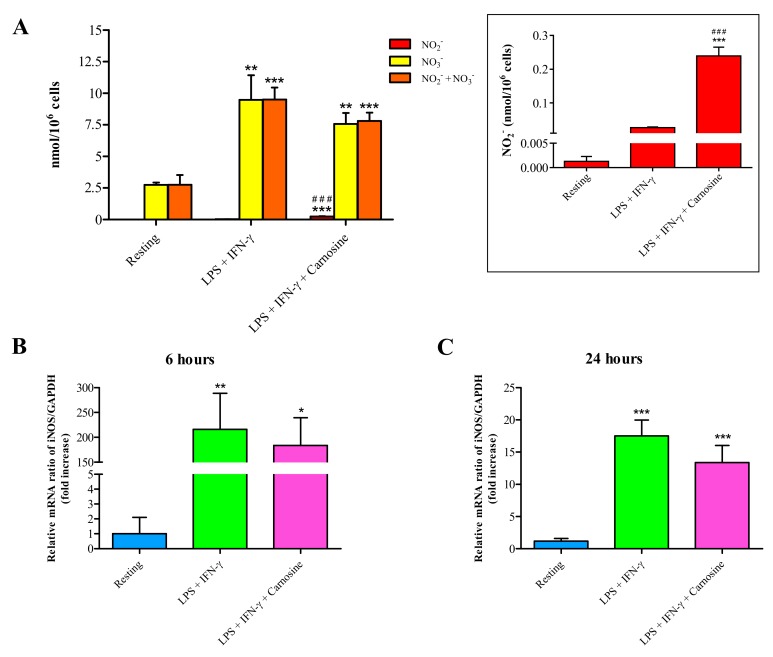
(**A**) Concentrations of NO metabolites (NO_2_^−^, NO_3_^−^,and NO_2_^−^ + NO_3_^−^) in resting (control) RAW 264.7 macrophages and in RAW 264.7 macrophages stimulated with LPS (100 ng/mL) + IFN-γ (600 U/mL), in the absence or in the presence of carnosine (20 mM). Values are means ± SD of three to four independent experiments and are expressed as nmol/million cells. Measurement of iNOS gene expression after (**B**) 6 h or (**C**) 24 h in cells treated as aforementioned. The abundance of iNOS mRNA was expressed relative to that of the housekeeping gene GAPDH. Values are means ± SD of three to four independent experiments. * *p* < 0.05 vs. resting; ** *p* < 0.01 vs. resting; *** *p* < 0.001 vs. resting; ^###^ *p* < 0.001 vs. LPS + IFN-γ.

To further investigate the carnosine’s ability to modulate the NO status, we performed experiments in which the formation of NO metabolites (nitrite, NO_2_^−^; nitrate, NO_3_^−^ and NO_2_^−^ + NO_3_^−^) as well as the expression of iNOS (the most representative enzyme implicated in NO production) were evaluated (Figure 2).

Figure 2A shows the formation of NO_2_^−^ and NO_3_^−^ as well as their sum (NO_2_^−^ + NO_3_^−^) in resting RAW 264.7 macrophages and in macrophages following the stimulation with LPS and IFN-γ, in the absence or in the presence of carnosine. NO_2_^−^ (0.001 ± 0.001 nmol/10^6^ cells) and NO_3_^−^ (2.75 ± 0.18 nmol/10^6^ cells) concentrations were quite low and low, respectively, in resting macrophages. As expected, the sum of NO metabolites was significantly increased following LPS + IFN-γ stimulation, both in the absence (+3.45 folds, *p* < 0.001 vs. resting) and in the presence (+2.84 folds, *p* < 0.001 vs. resting) of carnosine. The relative contribution of NO_2_^−^ and NO_3_^−^ to the aforementioned sum was greatly differed depending on the treatment. In fact, despite the absence of significant differences in the sum of the two metabolites, NO_2_^−^ (Figure 2A, insert) increased 9.19 folds in LPS + IFN-γ + carnosine stimulated macrophages (0.239 ± 0.026 nmol/10^6^ cells, *p* < 0.001 vs. resting) compared to LPS + IFN-γ only (0.027 ± 0.002 nmol/10^6^ cells). Concentration of NO_3_^-^ strongly increased from 2.75 ± 0.18 nmol/10^6^ cells in resting macrophages to 9.48 ± 1.96 nmol/10^6^ cells in LPS + IFN-γ treated macrophages (*p* < 0.001). The presence of carnosine caused a slight, but not significant, decrease in NO_3_^−^_,_ levels (7.57 ± 0.87 nmol/10^6^ cells) compared to LPS + IFN-γ treated macrophages. The gene expression of iNOS at 6 h (Figure 2B) dramatically increased by 216.02 ± 72.66 folds in LPS + IFN-γ treated macrophages (*p* < 0.001 vs. resting). A slight reduction occurred in the presence of carnosine (+183.76 ± 55.77 folds, *p* < 0.001 vs. resting). After 24 h stimulation without or with carnosine (Figure 2C), iNOS expression was still higher than that observed in resting cells (+18.36 ± 2.65 and +15.42 ± 4.62 folds in LPS + IFN-γ and LPS + IFN-γ + carnosine, respectively; *p* < 0.001 vs. resting).

To shed more light on the implication of iNOS in the observed increase in NO production in M1-activated macrophages, we also performed experiments employing two well-known iNOS inhibitors, namely L-NMMA and L-NAME. As shown in Supplementary Figure S2, iNOS activation is undoubtedly linked to the production of NO under our experimental conditions.

### 3.3. Carnosine Beneficially Affects High Energy Phosphates of LPS + IFN-γ-Stimulated RAW 264.7 Macrophages

**Figure 3:**
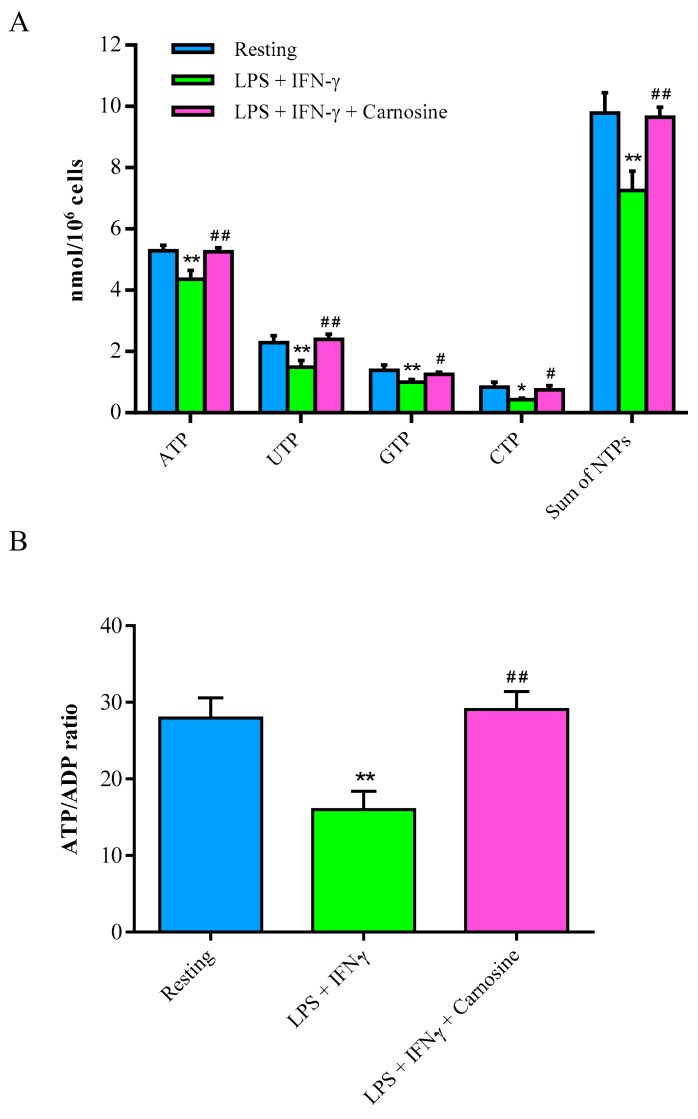
(**A**) Changes in nucleoside triphosphate concentrations (ATP, GTP, UTP, CTP and sum of nucleoside triphosphates), expressed as nmol/million cells, and (**B**) ATP/ADP ratio in resting (control) RAW 264.7 macrophages and in macrophages stimulated with LPS (100 ng/mL) + IFN-γ (600 U/mL), in the absence or in the presence of carnosine (20 mM). NTPs = nucleoside triphosphates. Values are means ± SD of three to four independent experiments. * *p* < 0.05 vs. resting; ** *p* < 0.01 vs. resting; ^#^ *p* < 0.05 vs. LPS + IFN-γ; ^##^ *p* < 0.01 vs. LPS + IFN-γ.

Figure 3A shows the effects of LPS + IFN-γ, in the absence or in the presence of carnosine, on the concentrations of high energy phosphates of RAW 264.7 macrophages.

After 24 h incubation with LPS + IFN-γ, a significant 18%, 29%, 26% and 50% decrease in the concentrations, respectively, of ATP, UTP, GTP, and CTP was recorded. Therefore, an overall 26% depletion in the sum of nucleoside triphosphates occurred in stimulated macrophages. Carnosine pre-treatment allowed maintaining both the concentrations of each compound and the high energy phosphate pool equal to those of control resting macrophages. Notably, concentration of ATP was fully restored by carnosine pre-treatment (5.28 ± 0.18, 4.35 ± 0.28 and 5.25 ± 0.13 nmol/10^6^ cells in resting, LPS + IFN-γ and LPS + IFN-γ + carnosine, respectively; *p* < 0.01 vs. both resting and LPS + IFN-γ + carnosine treated cells).

After 24 h in presence of the pro-inflammatory stimulus, 42% decrease of the ATP/ADP ratio (Figure 3B), which is considered an indicator of the mitochondrial phosphorylating capacity [42], was recorded (*p* < 0.01 vs. both resting and LPS + IFN-γ + carnosine treated cells). Carnosine pre-treatment restored ATP/ADP ratio values in macrophages, indicating unaltered mitochondrial function of energy production.

### 3.4. Carnosine Counterbalances the Changes in NAD^+^/NADH and NADP^+^/NADPH Ratio Induced by LPS + IFN-γ in RAW 264.7 Macrophages

**Figure 4:**
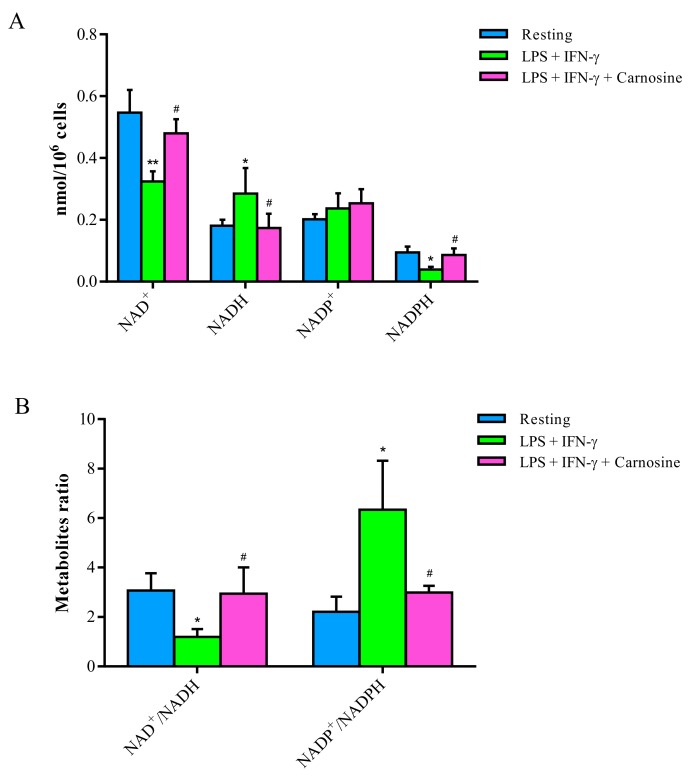
Changes in the concentrations of (**A**) nicotinic coenzymes (NAD^+^, NADH, NADP^+^ and NADPH) and (**B**) their corresponding ratio (NAD^+^/NADH and NADP^+^/NADPH) of RAW 264.7 macrophages incubated with LPS (100 ng/mL) + IFN-γ (600 U/mL), in the absence or in the presence of carnosine (20 mM). Values are means ± SD of three to four independent experiments. * *p* < 0.05 vs. resting; ** *p* < 0.01 vs. resting; ^#^ *p* < 0.05 vs. LPS + IFN-γ.

Data illustrated in Figure 4A show the beneficial effects of carnosine in preventing the imbalance of nicotinic coenzymes (NAD^+^, NADH, NADP^+^ and NADPH) caused by the incubation of macrophages with LPS + IFN-γ for 24 h.

The pro-inflammatory stimulus provoked a significant decrease in the concentrations of NAD^+^ (−41%) and NADPH (−59%), as well as an increase in the levels of NADH (+57%; *p* < 0.05 vs. both resting and LPS + IFN-γ + carnosine treated cells) and NADP^+^ (+18%; not significant). The presence of 20 mM carnosine in the incubation medium prevented the aforementioned changes, so that concentrations of reduced and oxidized nicotinic coenzymes did not differ from those determined in control resting macrophages.

The calculations of the oxidized to reduced nicotinic coenzyme ratio (Figure 4B) allowed it to evidence the influence of LPS + IFN-γ and of LPS + IFN-γ + carnosine on oxidative metabolism of glucose (NAD^+^/NADH), as well as on oxidative stress and antioxidant defenses (NADP^+^/NADPH), of stimulated macrophages.

A decrease by 61% of the NAD^+^/NADH ratio (suggesting increased glycolysis and potential mitochondrial dysfunction, and connected to the decrease in the ATP/ADP ratio) and increase by 186% of the NADP^+^/NADPH ratio (suggesting increase of NADPH-oxidase activity and decrease of pentose phosphate pathway and/or malic enzyme activity) were induced by the pro-inflammatory treatment (*p* < 0.05 vs. both resting and LPS + IFN-γ + carnosine), whilst no differences with respect to resting macrophages were observed in cells receiving LPS + IFN-γ + carnosine.

### 3.5. Carnosine Decreases LPS + IFN-γ-Induced Oxidative Stress in RAW 264.7 Macrophage Cells

**Figure 5:**
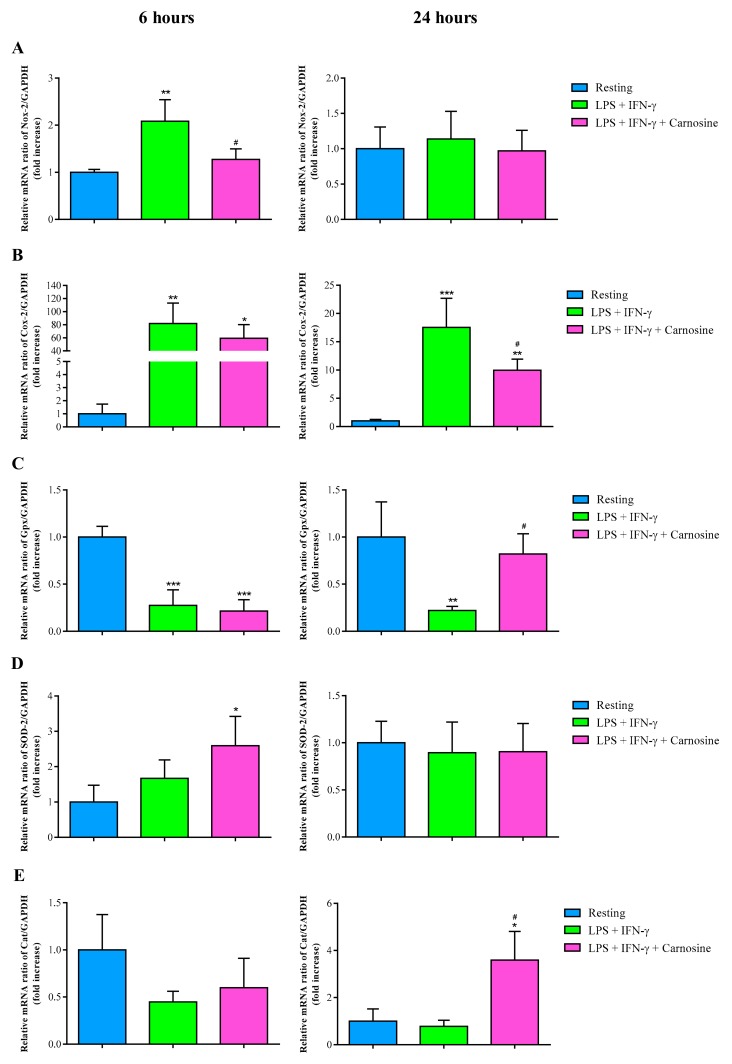
Measurement of (**A**) Nox-2, (**B**) Cox-2, (**C**) Gpx1, (**D**) SOD-2, and (**E**) Cat mRNA expression levels (qRT-PCR) in resting (control) RAW 264.7 macrophages and in macrophages stimulated with LPS (100 ng/mL) + IFN-γ (600 U/mL), in the absence or in the presence of carnosine (20 mM). The abundance of each mRNA of interest was expressed relatively to the abundance of GAPDH-mRNA. Values are means ± SD of three to four independent experiments. * *p* < 0.05 vs. resting; ** *p* < 0.01 vs. resting; *** *p* < 0.001 vs. resting; ^#^ *p* < 0.05 vs. LPS + IFN-γ.

In order to assess the ability of carnosine to counteract oxidative/nitrosative stress, we measured the gene expressions of ROS scavenger enzymes and of those generating ROS and RNS overflow in resting RAW 264.7 macrophages and in macrophages stimulated with LPS + IFN-γ, in the absence or in the presence of carnosine, for 6 h or 24 h. We found that the early increase of Nox-2 mRNA expression (Figure 5A), induced at 6 h by LPS + IFN-γ (*p* < 0.01 vs. resting) was abolished by 20 mM carnosine (*p* < 0.05 vs. LPS + IFN-γ).

No differences between the three different experimental conditions were observed at 24 h. Both at 6 h and 24 h, LPS + IFN-γ strongly increased Cox-2 mRNA expressions (+81.93 ± 31.22 folds at 6 h vs. resting, *p* < 0.01; +17.52 ± 5.14 folds at 24 h vs. resting, *p* < 0.001; Figure 5B). Carnosine significantly counteracted this phenomenon at 24 h only (*p* < 0.05 vs. LPS + IFN-γ). Stimulation of macrophages with LPS + IFN-γ remarkably decreased Gpx1 expression (Figure 5C), both at 6 h (*p* < 0.001 vs. resting) and 24 h (*p* < 0.01 vs. resting). Carnosine was able to restore Gpx1 expression to values equaling those found in controls only at 24 h (*p* < 0.05 vs. LPS + IFN-γ). The pro-inflammatory stimulus did not cause changes in the expressions of SOD-2 and Cat, although almost significant decrease in Cat mRNA expression was observed at 6 h (Figure 5D). Interestingly, the presence of carnosine during LPS + IFN-γ stimulation caused a significant two times increase in the gene expression of SOD-2 at 6 h (*p* < 0.05 vs. resting) and a significant three times increase in Cat gene expression at 24 h (*p* < 0.05 vs. resting and LPS + IFN-γ).

**Figure 6:**
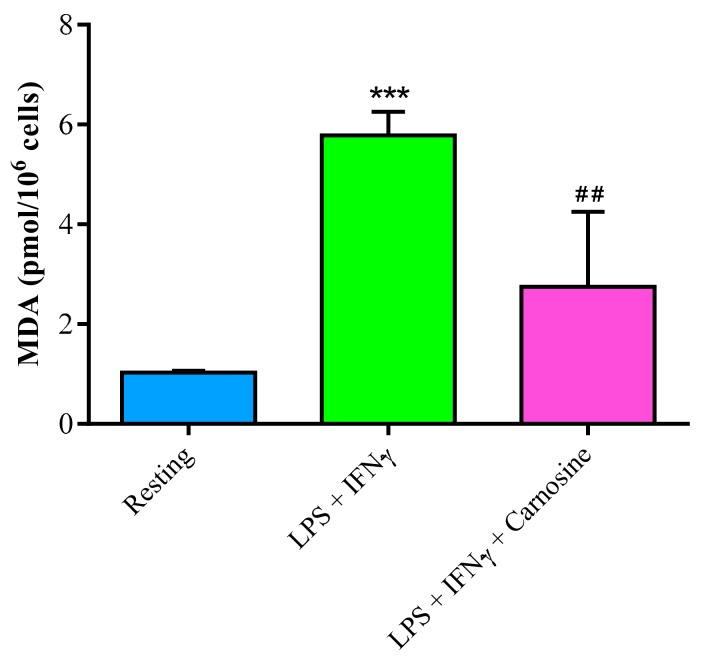
Concentration of MDA in resting (control) RAW 264.7 macrophages and in macrophages stimulated with LPS (100 ng/mL) + IFN-γ (600 U/mL), in the absence or in the presence of carnosine (20 mM). Values are means ± SD of three to four independent experiments. *** *p* < 0.001 vs. resting; ^##^ *p* < 0.01 vs. LPS + IFN-γ.

To further investigate the ability of carnosine in counteracting oxidative stress, we also measured the concentration of malondialdehyde (MDA; Figure 6), as a marker of ROS-mediated lipid peroxidation of polyunsaturated fatty acids of biological membranes phospholipids [43].

The concentration of MDA increased more than 5.5 times upon LPS + IFN-γ stimulation (1.02 ± 0.05 pmol/10^6^ cells in resting and 5.78 ± 0.46 pmol/10^6^ cells in stimulated macrophages; *p* < 0.001). Carnosine prevented the increase in MDA levels (2.75 ± 1.50 pmol/10^6^ cells; *p* < 0.01 vs. LPS + IFN-γ), indicating effective antioxidant activity.

### 3.6. Carnosine Decreases Inflammation Mediators and Increases TGF-β1 Expression

**Figure 7:**
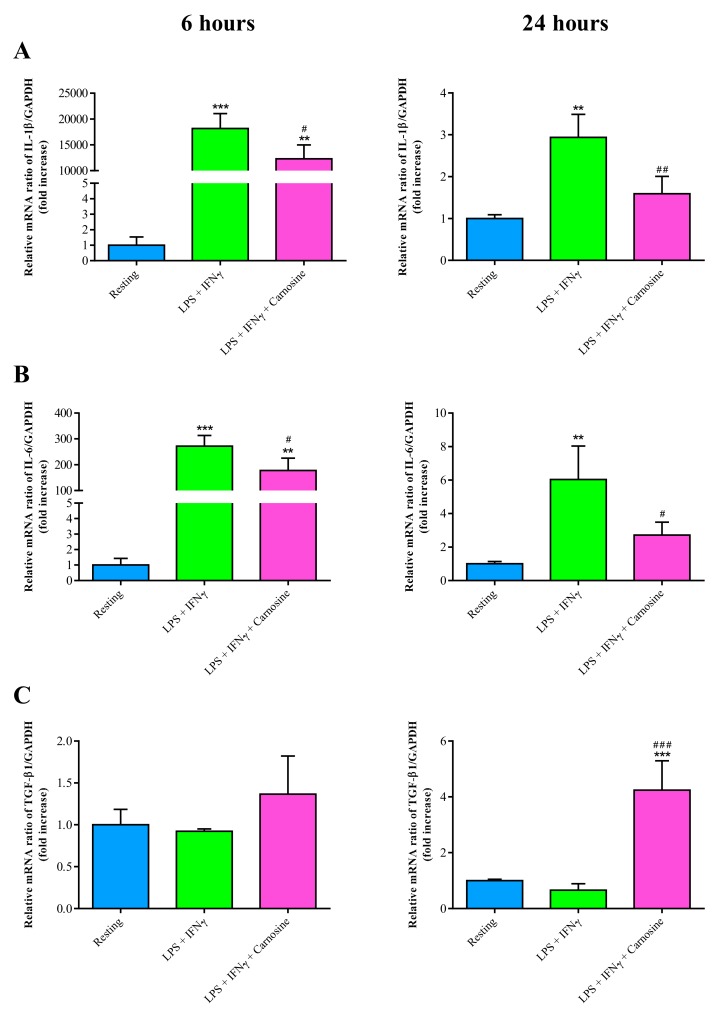
Gene expressions of IL-1β (**A**), IL-6 (**B**), and TGF-β1 (**C**) in resting (control) RAW 264.7 macrophages and in macrophages stimulated with LPS (100 ng/mL) + IFN-γ (600 U/mL), in the absence or in the presence of carnosine (20 mM), at 6 h and 24 h. The abundance of each mRNA was expressed relative to the abundance of GAPDH-mRNA. Values are means ± SD of three to four independent experiments. ** *p* < 0.01 vs. resting; *** *p* < 0.001 vs. resting; ^#^ *p* < 0.05 vs. LPS + IFN-γ; ^##^ *p* < 0.01 vs. LPS + IFN-γ; ^###^ *p* < 0.001 vs. LPS + IFN-γ.

Levels of the gene expression of IL-1β (Figure 7A) and IL-6 (Figure 7B) were strongly upregulated in macrophages following the stimulation with LPS + IFN-γ.

Specifically, the treatment of LPS + IFN-γ strongly increased IL-1β both at 6 h and 24 h. The presence of carnosine during the pro-inflammatory stimulus had significant beneficial effects at both time points (*p* < 0.05 at 6 h and *p* < 0.01 at 24 h). A similar scenario was observed for IL-6 (Figure 7B), with LPS + IFN-γ inducing hundreds and six fold increase in gene expression of IL-6 at 6 (*p* < 0.001 vs. resting) and 24 h (*p* < 0.01 vs. resting). As in the case of IL-1β, carnosine was able to significantly decrease the gene expression levels of IL-6 at both time points (*p* < 0.05 vs. LPS + IFN-γ). TGF-β1 mRNA expression, compared to that of resting macrophages, was unchanged at both 6 and 24 h by the treatment with LPS + IFN-γ. In accordance with the trend of the pro-inflammatory cytokines, the addition of carnosine during the challenge with LPS + IFN-γ did not cause a change in the gene expression of TGF-β1 at 6 h and produced an almost six times increase at 24 h (*p* < 0.001 vs. resting and LPS + IFN-γ), thus confirming that this compound is able to induce a late effective anti-inflammatory response. No significant differences were observed between LPS + IFN-γ and LPS + IFN-γ + Carnosine for IL-4 and IL-10 expression levels at both time points (data not shown).

### 3.7. Carnosine Strengthens the Antioxidant Machinery in RAW 264.7 Macrophages

**Figure 8:**
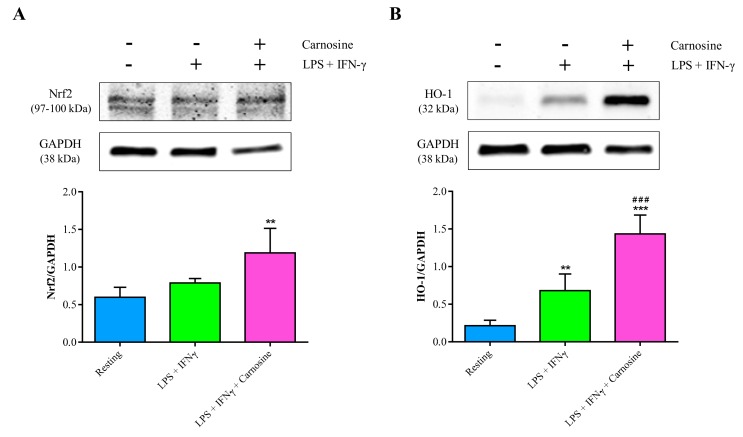
Representative immunoblots of (**A**) Nrf2 and (**B**) HO-1 in total protein extracts from resting (control) RAW 264.7 macrophages and in macrophages stimulated with LPS (100 ng/mL) + IFN-γ (600 U/mL), in the absence or in the presence of carnosine (20 mM). The abundance of each protein of interest was expressed relative to the abundance of the GAPDH protein, as an internal control. Histograms refer to the means ± SD of three to four independent experiments. ** *p* < 0.01 vs. resting; *** *p* < 0.001 vs. resting; ^###^ *p* < 0.001 vs. LPS + IFN-γ.

To further investigate the ability of carnosine in counteracting oxidative stress and/or reinforcing the antioxidant response in M1-polarized macrophages, we also considered its effect on the protein expression levels of Nrf2 and its downstream gene HO-1. Protein expression of Nrf2 was not significantly affected by the stimulation with LPS + IFN-γ, while it significantly increased when macrophages were pre-treated for 1 h with carnosine (*p* < 0.05 vs. resting; Figure 8A). Different effects were observed in the case of HO-1. In fact, the treatment of RAW 264.7 macrophages with LPS + IFN-γ induced a significant increase in HO-1 expression (+3.17 folds, *p* < 0.01 vs. resting), while carnosine pre-treatment strongly increased HO-1 concentration giving values 2.1 folds higher than LPS + IFN-γ-stimulated macrophages (*p* < 0.001) and 6.7 folds higher than those found in resting cells (*p* < 0.001; Figure 8B).

## 4. Discussion

Using an established in vitro model of inflammation (induced by LPS + IFN-γ) characterized by oxidative/nitrosative stress [7,33,44,45], in the present study we demonstrated that carnosine exerts potent anti-inflammatory and antioxidant activities, beneficially influencing gene expressions, protein expressions and energy metabolism of stimulated RAW 264.7 macrophages.

Macrophages play a pivotal role in initiating both inflammation and immune response [46]. When properly stimulated (e.g., using LPS), they undergo a series of processes involving the activation and release of pro-inflammatory and pro-oxidant mediators [47,48].

In our experiments, we stimulated RAW 264.7 cells with LPS (100 ng/mL) + IFN-γ (600 U/mL) concentrations properly chosen to avoid an increase in cell death during the exposure to these stimuli, but sufficient to produce changes in gene and protein expressions connected to inflammation and oxidative/nitrosative stress, as well as to cell energy metabolism. Molecular changes of our activated macrophages concerned modifications in the expression of genes encoding for proteins involved in NO production (increase in iNOS), ROS formation (increase in Nox-2), antioxidant defenses (decrease in Gpx) and pro-inflammatory stimulations (increase in Cox-2, IL-1β, and IL-6), indicating an overall M1 polarization [33]. Unlike Nrf2, the increase in protein expression of HO-1 was the only significant endeavor to counteract these changes. Notwithstanding HO-1 raise, stimulated macrophages did not only show increase of oxidative/nitrosative stress biomarkers (MDA, nitrite and nitrate), but also showed an imbalance of energy (decrease of ATP and other nucleoside triphosphates) and redox metabolism (alterations of NAD^+^/NADH and NADP^+^/NADPH ratio).

Whilst the decrease in the ATP/ADP ratio and the increase of the NAD^+^/NADH ratio suggested, respectively, reduced mitochondrial phosphorylating capacity [49] and compensatory increase of the glycolytic rate [50,51], the increase in the NADP^+^/NADPH ratio seems to be linked to the increase in Nox-2 levels, thus strongly supporting a major role of NADPH-oxidase (Nox-2) as a ROS generator during macrophage activation [52,53].

The switch to an inflammatory M1 phenotype of stimulated macrophages was effectively counteracted by the presence of 20 mM carnosine in the cell medium. This compound, having well-demonstrated minimal adverse effects [49,50] has been suggested as nutraceutical agents with a multimodal pharmacodynamic profile with potential applications in different pathological states such as inflammatory and neurodegenerative diseases [54,55].

In agreement with previous results, showing the ability of carnosine to indirectly counteract oxidative stress by enhancing the expression of members of the endogenous antioxidant system, including HO-1 and Nrf2 [56,57,58], under our experimental conditions we found that carnosine up-regulated both Nrf2 and, particularly, its downstream product HO-1 (Figure 8). Since the activity of HO-1 has been addressed as one of the main actors in the defense system towards oxidative/nitrosative stress [10,59,60,61,62], it is presumable that the carnosine-mediated positive HO-1 modulation contributes in part to the generalized decrease in metabolic indicators of sustained oxidative/nitrosative stress (sum of nitrite + nitrate, MDA; Figure 2 and Figure 6). On the other hand, carnosine effects on Nrf2 might have led stimulated macrophages to have lower expressions of Nox-2 and Cox-2 and concomitant higher expressions in canonical scavenger enzymes (Gpx, Cat, and SOD-2; Figure 5). All the above-mentioned results, showing an antioxidant activity of carnosine on activated macrophages, are in accordance with other studies in which the presence of this dipeptide protected brain macrophages and/or endothelial cells against cell death oxidative stress-induced [63,64].

According to this (Figure 2) and previous studies [31], we found that the presence of carnosine during macrophage stimulation with LPS + IFN-γ did not decrease the production of NO. Results of qRT-PCR of iNOS expression showed only a significant, but modest, effect of carnosine. However, the HPLC analysis of nitrite and nitrate, the stable end-products of NO, in the cell extracts allowed us to make new discoveries about the mechanisms underlying carnosine-induced NO degradation; in fact, our results clearly indicated that carnosine was able to produce a drastic 10 folds increase in nitrite (but not nitrate) concentration (Figure 2A, insert), thereby suggesting an increased degradation rate of NO into NO_2_^-^ mediated by carnosine.

This peculiar carnosine activity might certainly be of relevance for its potential therapeutic applications. It is well-known that excessive levels of iNOS-induced NO in macrophages are closely associated with different inflammatory diseases [57,58] and that when overproduced, NO reacts with superoxide ion generating peroxynitrite [22]. Carnosine, by accelerating NO degradation into NO_2_^−^, might efficiently decrease the levels of one of the substrates needed for the formation of peroxynitrite, diminishing risks of peroxynitrite-mediated damages to fundamental macromolecules [59]. In addition, NO overproduction in macrophages, induced by infectious agents, initiates apoptosis [17]; by decreasing NO bioavailability carnosine could prolong the immune response by preventing macrophages programmed cell death. It should also be considered that, during the last decade, several studies have shown the beneficial effects of NO_2_^−^ supplementation. Parthasarathy and Bryan showed that the use of foods or diets enriched with nitrite could have profound positive health benefits [60]. DeVan et al. provided evidence that ten weeks of sodium nitrite supplementation (80 or 160 mg/day) is able to significantly increase plasma nitrite concentrations acutely and chronically and was well tolerated with few side effects, improving endothelial function, and lessens carotid artery stiffening in middle-aged and older adults [65]. Hence, the carnosine activity on NO, leading to increase nitrite, would not cause any adverse effect but might rather represent an additional positive pharmacological effect of this natural compound.

Previous studies, using 500 ng/mL of LPS [66] or 1000 ng/mL LPS [67] to stimulate macrophages, showed significant changes in energy metabolism and mitochondrial functions. In the present study, using five and 10 times lower LPS concentrations than those used in the aforementioned studies, we found that 100 ng/mL LPS was sufficient to significantly perturb the energy and redox metabolism of macrophages (Figure 3 and Figure 4), whilst being completely free of any cytotoxic effect. The relevant finding was that all high-energy phosphates (ATP, UTP, GTP, and CTP) in stimulated RAW 246.7 were lower than corresponding values determined in resting control macrophages, indicating profound alterations of metabolic pathways and cycles devoted to the cell energy supply. Since the value of the NAD^+^/NADH ratio concomitantly halved, it is highly conceivable that a decrease in nucleoside triphosphates and in the ATP/ADP ratio (an index of mitochondrial phosphorylating capacity) were due to mitochondrial malfunctioning, particularly of the electron transfer chain coupled to oxidative phosphorylation, inducing a compensatory increase in glycolytic rate [50]. Interestingly, the pro-inflammatory and pro-oxidant stimulus produced an almost three times increase in the NADP^+^/NADPH ratio, strongly suggesting that this phenomenon (increase in the oxidized form of the coenzyme) is closely linked to the increase in the expression of NAPH-dependent Nox-2 (Figure 4A), one of the most active ROS generating system of activated macrophages [68].

From the metabolic point of view, the presence of 20 mM carnosine during macrophage stimulation was able to rescue both energy and redox metabolism. Particularly, carnosine allowed stimulated macrophages to maintain correct mitochondrial functions, as evidenced by ATP/ADP and NAD^+^/NADH ratio equal to those of controls and evidencing correct mitochondrial phosphorylating capacity and functioning of the electron transport chain (Figure 3 and Figure 4 ). These results are in line with previous studies showing that carnosine has beneficial effects on energy metabolism during periods of cell sufferance induced by various stimuli [69,70].

Restoration of cell metabolism by carnosine was accompanied by the downregulation of the expression of two of the major pro-inflammatory cytokines, namely IL-1β (Figure 7A) and IL-6 (Figure 7B), having a crucial role in initiating the inflammatory process. Different studies have linked the deleterious effects of IL-1β with different pathological conditions, including type 1 diabetes [71,72], gout [73], Alzheimer’s disease [74] and many auto-inflammatory disorders [75]. Pre-clinical studies in rodents have demonstrated a robust activation of both astrocytes and microglia following IL-1β injection or induction [74]. Additionally, IL-1β is capable of triggering further increases in its own expression and the production of additional pro-inflammatory cytokines, such as IL-6 [76]. The up-regulation of IL-6 has been reported in different neuroinflammatory and neurodegenerative disorders of the central nervous system [77], pulmonary hypertension [78], metabolic syndrome [79], depression [80] as well as cancer [81]. Altogether, our results and those reported in the aforementioned studies underline the potentially beneficial roles of carnosine administration in counteracting inflammatory phenomena characterized by elevated levels of IL-1β and/or IL-6.

In addition to IL-1β and IL-6, carnosine pre-treatment was able to up-regulate the expression of the TGF-β1 gene in LPS + IFN-γ-stimulated macrophages (Figure 7C). TGF-β1 is an anti-inflammatory cytokine of key importance in the control of cell growth and differentiation as well as for the immune response [82,83]. It has been shown in vivo that the administration of TGF-β1 is able to protect against Aβ-induced neuroinflammation and neurodegeneration [84,85,86], whilst mutations of the TGF-β1 gene in mice lead to death from multifocal inflammation and autoimmune disorders in internal organs [87,88]. Additionally, a significant deficit of TGF-β1, paralleling memory deficits and depressive-like phenotype, has been found in the hippocampus of Aβ-injected mice [89]. Very recently, the ability of carnosine to protect brain macrophages and neurons against Aβ-induced cell toxicity by increasing TGF-β1 expression and secretion has been proposed [23].

Lastly, with the aim to reinforce the importance of carnosine in the “antioxidant scenario”, we evaluated the ability of this molecule to modulate Nrf2 and HO-1, both of them playing a pivotal role in the intracellular antioxidant system [90,91]. Carnosine significantly up-regulated both proteins, giving expression values higher than those observed in resting and LPS + IFN-γ-stimulated cells (*p* < 0.001 for both of them) for HO-1 and higher than resting (*p* < 0.01) for Nrf2 (Figure 8). These results are in agreement with previous studies showing the ability of carnosine to indirectly counteract oxidative stress by enhancing the expression of members of the endogenous antioxidant system, including HO-1 and Nrf2 [56,57,58]. According to the role of Nrf2 signaling pathway [92], our data suggest that carnosine-mediated induction of HO-1 and Nrf2 may contribute to the inhibition of oxidative and inflammatory responses in RAW 264.7 macrophages. This effect may certainly be achieved due to the carnosine-induced rescue of Gpx1 and increase of SOD-2 and Cat gene expressions.

The above-mentioned findings provide us with a solid foundation for a further investigation of the multimodal mechanism of action of carnosine in animal model characterized by both inflammation and oxidative stress such as depression and/or AD.

## 5. Limitations of the Study

The present study clearly shows the antioxidant and anti-inflammatory features exerted by carnosine on activate macrophages. Despite that, an additional mechanism working along with the well characterized “Carnosine-NO axis” should be identified in order to give a high-level overview of the process. Furthermore, this study was conducted in one particular cell line under specific conditions (M1 activation in absence of cell toxicity), and then caution should be exercised in making generalizations across different cell lines and/or protocols.

## 6. Conclusions

In the present study we provided strong evidence about carnosine ability to modulate pro-oxidant and pro-inflammatory activities of activated (M1) macrophages. Our results clearly show a dual antioxidant role of carnosine highlighted by its ability to reinforce the antioxidant machinery (Nrf2, HO-1, and ROS scavenger enzymes up-regulation), simultaneously decreasing the expression of pro-oxidant enzymes (Nox-2 and Cox-2) and lipid peroxidation (MDA). The presence of carnosine was also able to counteract the imbalance in energy and redox metabolism observed in M1 macrophages by restoring nucleoside triphosphates and counterbalancing the changes in ATP/ADP, NAD^+^/NADH and NADP^+^/NADPH ratio obtained by LPS + IFN-γ induction. We again showed the peculiar carnosine ability to increase NO degradation rate into its non-toxic end-products, providing deeper insights into the carnosine-mediated transformation into NO_2_^−^. Carnosine also exerted a strong anti-inflammatory activity by down-regulating the LPS + IFN-γ-induced expression of IL-1β and IL-6, simultaneously up-regulating the expression of the anti-inflammatory cytokine TGF-β1.

All the aforementioned carnosine effects suggest a preclinical and clinical relevance of these results in light of potential therapeutic use of this naturally occurring dipeptide in counteracting pro-oxidant and pro-inflammatory phenomena as a novel pharmacological tool for the treatment of diseases characterized by elevated levels of oxidative stress and inflammation.

## Figures and Tables

**Table 1:** The list of primers used for quantitative real-time PCR (qRT-PCR).

Official Name ^#^	Official Symbol	Alternative Titles/Symbols	Detected Transcript	Amplicon LENGTH	Cat. No. ^§^
nitric oxide synthase 2, inducible	Nos2	iNOS; Nos-2; Nos2a; i-NOS; NOS-II; MAC-NOS	NM_010927	118 bp	QT00100275
cytochrome b-245, beta polypeptide	Cybb	Cgd; Cyd; Nox2; C88302; gp91-1; gp91phox; CGD91-phox	NM_007807 XM_006527565	146 bp	QT00139797
superoxide dismutase 2, mitochondrial	Sod2	MnSOD; Sod-2	NM_013671	159 bp	QT00161707
catalase	Cat	Cas1; Cs-1; Cas-1; 2210418N07	NM_009804 XM_006498624	121 bp 121 bp	QT01058106
glutathione peroxidase 1	Gpx1	Gpx; CGPx; GPx-1; GSHPx-1; AI195024; AL033363	NM_008160	133 bp	QT01195936
prostaglandin-endoperoxide synthase 2	Ptgs2	COX2; Cox-2; PES-2; PHS-2; Pghs2; TIS10; PGHS-2; PHS II; gripghs	NM_011198	95 bp	QT00165347
interleukin 1 beta	Il1b	Il-1b; IL-1beta; IL-1β	NM_008361 XM_006498795	150 bp 682 bp	QT01048355
interleukin 6	Il6	Il-6	NM_031168	128 bp	QT00098875
interleukin 4	Il4	Il-4; BSF-1	NM_021283	132 bp	QT02418311
interleukin 10	Il10	CSIF; Il-10	NM_010548	103 bp	QT00106169
transforming growth factor, beta 1	Tgfb1	Tgfb; Tgfb-1; TGFbeta1; TGF-beta1	NM_011577	145 bp	QT00145250
glyceraldehyde-3-phosphate dehydrogenase	Gapdh	Gapd	NM_008084 XM_001003314 XM_990238 NM_001289726	144 bp	QT01658692

^#^https://www.ncbi.nlm.nih.gov/gene/; ^§^https://www.qiagen.com/it/shop/pcr/real-time-pcr-enzymes-and-kits/two-step-qrt-pcr/quantitect-primer-assays/.

## Supplementary Materials

Supplementary Materials can be found at https://www.mdpi.com/1422-0067/21/3/776/s1. Figure S1. The protocol and methods employed to evaluate cytotoxicity (MTT assay), energy metabolism (HPLC), gene expressions (high-throughput real-time PCR (qRT-PCR)), protein expressions (western blot) and nitric oxide production (qRT-PCR, HPLC), in activated macrophages. Figure S2. Extracellular concentrations of nitrite in resting RAW 264.7 macrophages and in macrophages stimulated for 24 h with LPS + IFN-γ (100 ng/mL + 600 U/mL, respectively), in the absence or in the presence of L-NMMA (500 µM) or L-NAME (1 mM) (1 h pretreatment).
