# Supplementary material for: Modulation of Pro-Oxidant and Pro-Inflammatory Activities of M1 Macrophages by the Natural Dipeptide Carnosine
Source: Int J Mol Sci. 2020 Jan 25;21(3):776. doi: 10.3390/ijms21030776 (PMC7038063; doi:10.3390/ijms21030776)
Supplement: Supplementary file 1 [file ijms-21-00776-s001.pdf]

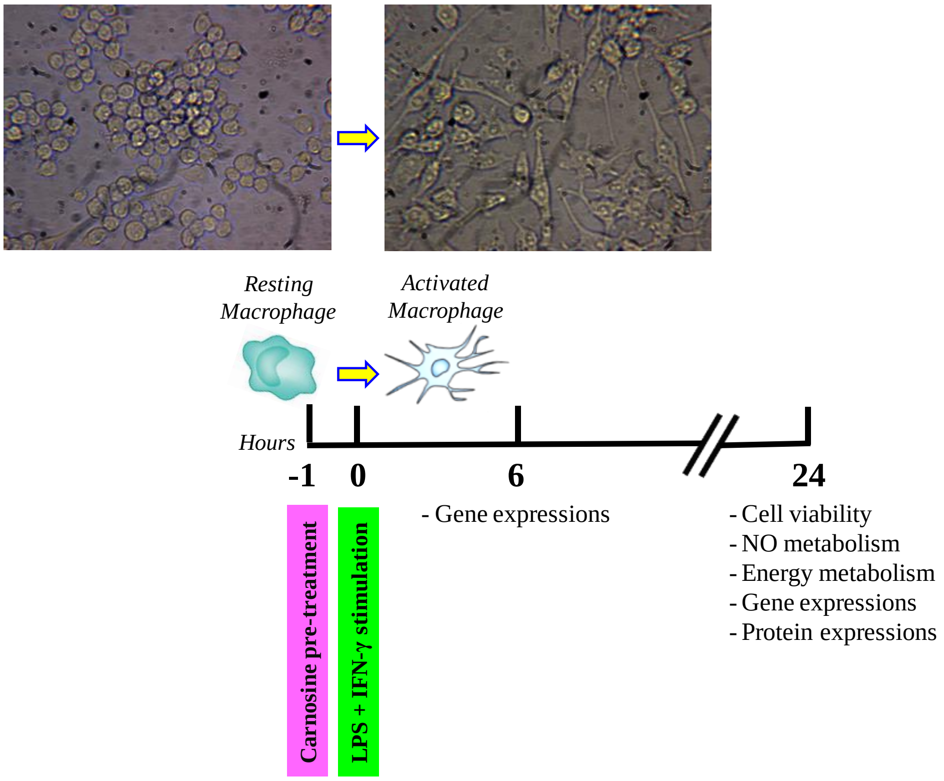

Figure S1. The protocol and methods employed to evaluate cell viability (MTT assay), energy metabolism (HPLC), gene expressions (high-throughput real-time PCR (qRT-PCR)), protein expressions (western blot) and nitric oxide production (qRT-PCR, HPLC), in activated macrophages.

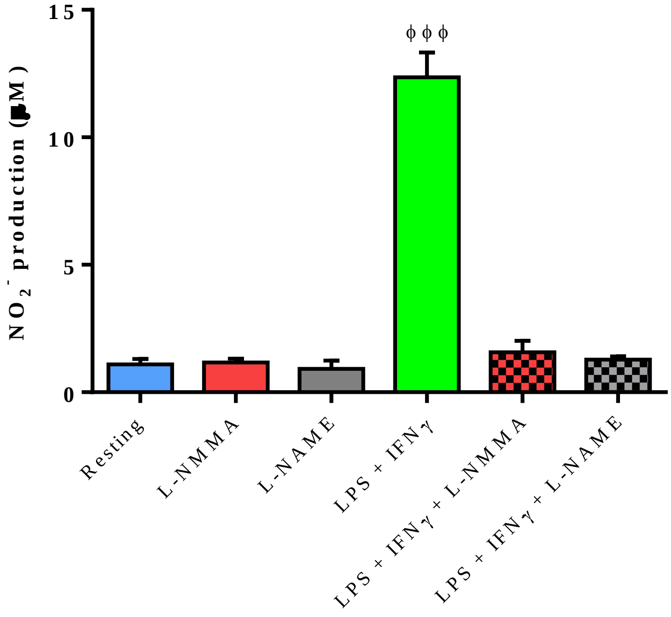

Figure S2. Extracellular concentrations of nitrite in resting RAW 264.7 macrophages and in macrophages stimulated for 24 h with LPS + IFN-γ (100 ng/mL + 600 U/mL, respectively), in the absence or in the presence of L-NMMA (500 µM) or L-NAME (1 mM) (1 h pretreatment). L-NMMA = N^G-Monomethyl-L-arginine; L-NAME = N^{ω}-*Nitro-l-Arginine Methyl Ester*. Values are means ± SD of three independent experiments. $^{\Phi\Phi\Phi}p < 0.001$ vs. all other treatments.
